# Opioid-Related Hospitalization and Its Association With Chronic Diseases: Findings From the National Inpatient Sample, 2011–2015

**DOI:** 10.5888/pcd16.190169

**Published:** 2019-11-27

**Authors:** Janani Rajbhandari-Thapa, Donglan Zhang, Heather M Padilla, Sae Rom Chung

**Affiliations:** 1 Department of Health Policy and Management, College of Public Health, University of Georgia, Athens, Georgia; 2 Department of Health Promotion and Behavior, College of Public Health, University of Georgia, Athens, Georgia; 3 Department of Financial Planning, Housing, and Consumer Economics, College of Family and Consumer Sciences, University of Georgia, Athens, Georgia

## Abstract

Chronic disease and opioid-related hospitalizations in the United States are increasing. We analyzed nationally representative data on patients aged 18 years or older from the 2011–2015 National Inpatient Sample to assess the association between opioid-related hospitalization and chronic diseases. We found that most patients with opioid-related hospitalization were white, aged 35–54 years, in urban hospitals, and had 2 or more comorbid conditions. Patients with 2 or more chronic conditions accounted for more than 90% of opioid-related hospitalizations in all years. The results suggest a need for targeted interventions to prevent opioid misuse in patients with multiple chronic conditions.

**Summary**

**What is already known on this topic?**

Patients who take addictive pain relievers often have chronic conditions.

**What is added by this report?**

From 2011 to 2015, more than 90% of US opioid-related hospitalizations were among patients with 2 or more chronic diseases. The trend in opioid-related hospitalization among patients with multiple chronic diseases is increasing and noteworthy.

**What are the implications for public health practice?**

Multiple chronic diseases are associated with opioid-related hospitalizations, indicating that prevention of opioid-related abuse and overdose among patients with multiple chronic conditions requires alternatives to opioid prescription, when possible.

## Objective

Patients with chronic conditions such as cancer, stroke, asthma, and obesity often experience chronic pain and have a higher likelihood of receiving one or more opioid prescriptions (1). In this study, we examined whether the prevalence of opioid-related hospitalization was associated with chronic diseases among inpatients. This study is the first to examine the prevalence of opioid-related hospitalization and chronic disease among patients admitted to community hospitals. Opioid-related inpatient stays (2) have increased, mainly due to opioid abuse, addiction, poisoning, and dependence, resulting from misuse of opioid pain relievers that are often originally prescribed (3) to treat chronic pain.

## Methods

In this cross-sectional study, we analyzed data on patients aged 18 years or older from the National Inpatient Sample (NIS) from January 1, 2011, to September 30, 2015. Claims occurring from October through December of 2015 were not included because of the transition from the International Classification of Diseases, 9th Revision, Clinical Modification (ICD-9-CM) to the 10th revision (ICD-10-CM) coding system on October 1, 2015. We included arthritis, spinal disease, asthma, liver disease, stroke, cancer, and obesity because these diseases are most likely to be prescribed opioids in the internal and family medicine specialties. Family medicine (21.8%) and internal medicine (17.6%) reportedly write the most opioid prescriptions, followed by physical medicine/rehabilitation, anesthesiology/pain, hematology/oncology, and neurology (4–6). Relationships between asthma and opioid abuse and dependence (7) and between chronic liver disease and opioid use (8) have been established, as has a relationship between stroke and opioid abuse among young stroke patients (9). Cancer patients rely on opioid medications for relief from cancer pain (10). Obesity was included because of its significant association with chronic pain (11). Using the NIS sample, we identified 3,239,136 opioid-related hospitalization cases for from January 1, 2011, to September 30, 2015.

We combined opioid dependence and unspecified use, adverse effects of opioids, opioid abuse, and opioid poisoning to create a single variable, *opioid-related hospitalization* (specific ICD-9-CM codes are available in Appendices A and B). The data were weighted by using sampling weights provided by the NIS Healthcare Cost and Utilization Project (12). For creating national estimates for the prevalence of opioid-related hospitalizations and all analyses, we implemented Stata’s trend weight (TRENDWT) for 2011 and discharge weight (DISCWT) from 2012 through 2015 (StataCorp LLC). Because of the redesign of NIS data in 2012, we used TRENDWT for 2011, which allowed the national estimates for trend analysis in 2011 to be consistent with data from 2012–2015. To use trend weight, we merged the trend weight file (www.hcup-us.ahrq.gov/db/nation/nis/trendwghts.jsp) into the original NIS 2011 data. We analyzed the trends in opioid-related hospitalizations by type of chronic disease for 2011–2015 and used the Pearson χ^2^ test to test for significance. Significance was set at *P* < .05.

## Results

The highest total number of opioid-related hospitalization cases was in 2014 (n = 704,670) (Table 1). From 2011 to 2015, the highest prevalence of opioid-related hospitalization was among patients aged 35–54 years (5-year average, 37%), followed by those aged 18–34 years. The percentages of men and women with opioid-related hospitalizations were approximately equal. The highest prevalence of opioid-related hospitalization was among white patients (5-year average, 72%), and the second-highest was among black patients (5-year average, 15%). The prevalence was higher in urban hospitals (5-year prevalence range, 58%–64%) than in rural hospitals (5-year prevalence range, 36%–42%). The prevalence was also higher among patients who used Medicaid and Medicare than among those who did not. The prevalence of opioid-related hospitalization was highest (94%) among patients with 2 or more comorbid conditions. In 2014 and 2015, as many as 95% of the patients hospitalized for opioid-related causes had 2 or more comorbid conditions.

**Table 1:** Prevalence of Opioid-Related Hospitalizations Among Adults Aged 18 or Older, National Inpatient Sample, 2011–2015^a^

Characteristic	Year^b^				
	2011 (n = 639,586)	2012 (n = 655,520)	2013 (n = 664,480)	2014 (n = 704,670)	2015 (n = 574,880)
**Age, y**					
18–34	29.35	30.30	30.37	29.98	29.22
35–54	39.51	38.23	37.07	36.29	35.97
55–64	15.86	16.50	17.06	17.60	18.37
≥65	15.29	14.97	15.50	16.12	16.44
**Sex**					
Male	49.73	49.34	49.02	49.15	49.63
Female	50.27	50.66	50.98	50.85	50.37
**Race/ethnicity**					
White	70.76	71.26	72.67	73.16	72.99
Black	16.30	15.36	14.22	14.12	14.44
Hispanic	8.33	8.70	8.79	8.30	8.38
Asian	0.69	0.78	0.75	0.84	0.88
Native American	0.65	0.70	0.65	0.74	0.77
Other	3.27	3.20	2.84	2.84	2.59
**No. of comorbidities**					
None	0.69	0.57	0.54	0.39	0.40
One	5.39	5.41	4.97	4.49	4.23
Two or more	93.91	94.02	94.48	95.12	95.37
**Rural/urban status**					
Rural	35.59	40.81	42.04	41.57	41.71
Urban	64.41	59.19	57.96	58.43	58.29
**Payer**					
Medicaid	30.74	31.61	31.29	37.66	39.30
Medicare	30.20	30.62	31.56	31.85	31.53
Private	21.22	21.06	20.72	19.62	19.61
Self-pay	13.35	12.19	12.02	7.80	6.45
Other	4.49	4.52	4.41	3.08	3.11

a Data are percentages, per 100,000 hospitalizations; sampling weight adjusted in all statistics.

b Values for n indicate the total number of opioid-related hospitalizations for the year.

The prevalence of opioid-related hospitalizations significantly increased among patients with cancer, stroke, obesity, asthma, liver disease, and arthritis across all years (Table 2). The most prevalent chronic disease significantly associated with opioid-related hospitalization was asthma, followed by obesity and liver disease. There was a significant association between opioid-related hospitalization and cancer, stroke, obesity, asthma, and liver disease every year of the study period, with the exception of cancer in 2013 and obesity in 2015. The association between arthritis and opioid-related hospitalizations was significant only in 2014 and 2015, and the association between spinal diseases and opioid-related hospitalizations was significant only in 2012 and 2015.

**Table 2:** Prevalence of Opioid-Related Hospitalizations Among Adults Aged 18 or Older, by Chronic Disease, National Inpatient Sample, 2011–2015^a^

Chronic Disease	Year					*P* Value^b^
	2011	2012	2013	2014	2015	
Cancer	1.11^c^	1.16^c^	1.32	1.40^c^	1.58^c^	<.001
Stroke	0.90^c^	0.93^c^	1.04^c^	1.04^c^	1.07^c^	<.001
Obesity	8.18^c^	8.92^c^	9.67^c^	10.21^c^	10.80	<.001
Asthma	10.77^c^	11.18^c^	10.93^c^	11.49^c^	11.91^c^	<.001
Liver Disease	4.76^c^	4.77^c^	5.12^c^	5.21^c^	5.35^c^	<.001
Spinal Disease	0.83	0.75^d^	0.77	0.77	0.80^c^	.09
Arthritis	1.65	1.67	1.73	1.83^d^	1.96^c^	<.001

a Data are percentages, per 100,000 hospitalizations; sampling weight adjusted in all statistics.

b Trend analysis was calculated by using χ^2^ test.

c *P* < .001. *P* values calculated by using test.

d *P* < .05. *P* values calculated by using test.

## Discussion

We found that more than 90% of opioid-related hospitalizations were among patients with 2 or more chronic diseases and that the trend in opioid-related hospitalization among patients with chronic diseases is increasing. This finding is salient given that 1 in 4 US adults are living with 2 or more chronic diseases (13). Both the opioid crisis and rising rates of chronic disease have been described as epidemics; however, they are currently being treated separately. The US Department of Health and Human Services’ 5-point strategy to combat the opioid crisis focuses on the treatment of addiction, which is important for addressing the epidemic in the short term (14). A population-level approach to preventing and treating chronic diseases is also needed. Health services must address the 2 issues — opioid misuse and management of chronic disease — simultaneously.

Using alternative approaches to pain management is one public health strategy for addressing the opioid crisis (15) and is consistent with the Centers for Disease Control and Prevention’s (CDC’s) Guideline for Prescribing Opioids for Chronic Pain (16). Based on our findings, this approach may be particularly important in patients with multiple chronic diseases. Consistent with the CDC guideline, nonpharmacologic treatment including exercise therapy and cognitive behavioral therapy, as well as multimodal therapies combining exercise therapy with psychologically based approaches, should be used to decrease pain and improve functional ability (16). When opioids are used for pain management, patients with multiple chronic diseases may require close monitoring and additional education on risks of misuse.

This study has several limitations. Because we analyzed data from 2011 to 2015, our findings are not generalizable to more recent years. Our analysis was cross-sectional and examined the associations between opioid-related hospitalization and chronic diseases without controlling for covariates, which prevents us from drawing causal conclusions about the direction of the observed relationship. Furthermore, the nature of the data did not allow us to explore why the associations were not significant in some years. Opioid prescriptions for patients with arthritis have increased in recent years, so that could be an explanation for the strong correlation we found (17). Finally, we studied a small number of chronic diseases and did not assess any diseases related to mental health.

Despite these limitations, our findings show an increasing trend in opioid-related hospitalization among patients with chronic diseases and a high prevalence of opioid-related hospitalization among patients with multiple chronic conditions. The observed association warrants further research to determine causal inference. Results from this study can help clinicians develop strategies to prevent opioid misuse in patients with multiple chronic conditions and inform strategies for addressing both the opioid epidemic and rising rates of chronic disease.

## Appendix A *International Classification of Diseases, 9th Revision, Clinical Modification* (*ICD-9-CM*) Diagnosis Codes Used to Identify Opioid-Related Hospitalization

**Table:**

Hospitalization Type	*ICD-9-CM* Code
Opioid dependence and unspecified use	304.00, 304.01, 304.07, 304.71
Adverse effects of opioids	E935.1, E935.2, E940.1
Opioid abuse	305.50, 305.51, 305.52
Opioid poisoning	965.00, 965.01, 965.02, 965.09, 970.1, E850.0, E850.1, E850.2

## Appendix B *International Classification of Diseases, 9th Revision, Clinical Modification* (*ICD-9-CM*) Diagnosis Codes Used to Identify Comorbidities

**Table:**

Comorbidity	*ICD-9-CM* Code
**Cancer**	
Buccal cavity and pharynx	140.00 to 149.00
Digestive system	150.00 to 159.00
Respirator system	160.00 to 165.00
Bones and joints	170.00
Soft tissue including heart	171.00
Skin	172.00 to 173.00
Breast	174.00 to 175.00
Kaposi’s sarcoma	176.00
Female genital system	179.00, 180.00 to 184.00
Male genital system	185.00 to 187.00
Urinary system	188.00 to 189.00
Eye and orbit	190.00
Brain and other nervous system	191.00 to 192.00
Endocrine system	164.00, 193.00 to 194.00
Ill-defined	195.00 to 196.00, 199.00, 202.00
Lymphoma	200.00 to 202.00, 202.10, 202.20, 202.80, 202.90
Multiple myeloma	203.00, 203.80
Leukemia	202.40, 203.10, 204.00 to 208.00
Benign neoplasms	210.00 to 229.00
Carcinoma in situ	230.00 to 234.00
Neoplasms of uncertain behavior	235.00 to 238.00
Neoplasms of unspecified nature	239.00
**Stroke**	
Occlusion and stenosis of basilar artery	433.01, 433.10, 433.11, 433.21, 433.31, 433.81, 433.91
Cerebral thrombosis with/without cerebral infarction	434.00, 434.01, 434.11, 434.91
Acute, but ill-defined, cerebrovascular	436.00
Subarachnoid hemorrhage	430.00
Intracerebral hemorrhage	431.00
**Asthma**	
Extrinsic asthma	493.00, 493.01, 493.02
Intrinsic asthma	493.10, 493.11, 493.12
Chronic obstructive asthma	493.20, 493.21, 493.22
Other forms of asthma	493.81, 493.82
Asthma unspecified	493.90, 493.91, 493.92
**Liver disease**	
Acute and subacute necrosis of liver	570.0
Chronic liver disease and cirrhosis	571.0, 571.1, 571.2, 571.3, 571.4, 571.5, 571.6, 571.8, 571.9
Liver abscess	572.0. 572.1, 572.2, 572.3, 572.4, 572.8
Other disorders of liver	573.0, 573.1, 573.2, 573.3, 573.4, 573.5, 573.6, 573.8, 573.9
**Arthritis**	
Psoriatic arthritis	696.0
Diffuse disease of connective tissue	710.0. 710.1. 710.2. 710.3, 710.4, 710.5, 710.8, 710.9
Infectious arthropathies	711.0, 711.1, 711.2, 711.3, 711.4, 711.5, 711.6, 711.7, 711.8, 711.9
Crystal arthropathies	712.1, 712.2, 712.3, 712.8, 712.9
Rheumatoid arthritis	714.0. 714.2, 714.3, 714.4, 714.8, 714.9
Osteoarthritis and allied disorders	715.0, 715.1, 715.2 715.3, 715.8, 715.9
Other/unspecified arthropathies	716.0, 716.1, 716.2, 716.3, 716.5, 716.6, 716.8, 716.9
**Obesity**	
Obesity	278.00
Morbid obesity	278.01
Overweight	278.02
Obesity hypoventilation syndrome	278.03
**Spinal disease**	
Spondylosis	721.0
Intervertebral disc disorder	722.0
Other cervical disorders	723.0
Low back pain	724.0
